# Assisted injection in outdoor venues: an observational study of risks and implications for service delivery and harm reduction programming

**DOI:** 10.1186/1477-7517-7-6

**Published:** 2010-03-19

**Authors:** Elisa Lloyd-Smith, Beth S Rachlis, Diane Tobin, Dave Stone, Kathy Li, Will Small, Evan Wood, Thomas Kerr

**Affiliations:** 1 British Columbia Centre for Excellence in HIV/AIDS, St. Paul's Hospital, Vancouver, British Columbia, Canada; 2 Injection Support Team, Vancouver Area Network of Drug Users, Vancouver, British Columbia, Canada

## Abstract

**Background:**

Assisted injection and public injection have both been associated with a variety of individual harms including an increased risk of HIV infection. As a means of informing local IDU-driven interventions that target or seek to address assisted injection, we examined the correlates of receiving assistance with injecting in outdoor settings among a cohort of persons who inject drugs (IDU).

**Methods:**

Using data from the Vancouver Injection Drug Users Study (VIDUS), an observational cohort study of IDU, generalized estimating equations (GEE) were performed to examine socio-demographic and behavioural factors associated with reports of receiving assistance with injecting in outdoor settings.

**Results:**

From January 2004 to December 2005, a total of 620 participants were eligible for the present analysis. Our study included 251 (40.5%) women and 203 (32.7%) self-identified Aboriginal participants. The proportion of participants who reported assisted injection outdoors ranged over time between 8% and 15%. Assisted injection outdoors was independently and positively associated with being female (Adjusted Odds Ratio (AOR) = 1.74, 95% Confidence Intervals (CI): 1.21-2.50), daily cocaine injection (AOR = 1.70, 95% CI: 1.29-2.24), and sex trade involvement (AOR = 1.44, 95% CI: 1.00-2.06) and was negatively associated with Aboriginal ethnicity (AOR = 0.58, 95% CI: 0.41-0.82).

**Conclusions:**

Our findings indicate that a substantial proportion of local IDU engage in assisted injecting in outdoor settings and that the practice is associated with other markers of drug-related harm, including being female, daily cocaine injecting and sex trade involvement. These findings suggest that novel interventions are needed to address the needs of this subpopulation of IDU.

## Background

The injection of illicit substances is associated with an array of harms. The transmission of bacterial and viral infections and risk of overdose persists in a range of settings despite considerable differences in drugs consumed and local injecting practices [1]. In response, a range of interventions have been developed to target unsafe injecting [1]. However, unsafe injection often continues despite a growing availability of interventions that specifically target these problems.

Supervised injection facilities (SIF) are a novel form of intervention that typically involve providing a hygienic environment where persons who inject drugs (IDU) can inject under the supervision of health care professionals [2]. North America's first SIF is situated in Vancouver, Canada's Downtown Eastside (DTES) [2], a neighbourhood characterized by extreme poverty, high crime, homelessness, poor housing, and high rates of alcohol and drug abuse [3]. Research on the SIF has demonstrated success in attracting high-risk injectors [4], as well as improvements in safer injecting practices such as reduced levels of syringe sharing [5]. However, as with many other interventions that target unsafe injecting, concerns regarding barriers to SIF use remain. In particular, assisted injection, or being physically injected by someone else, is prohibited [6]. The prohibition on assisted injection at the SIF is structured by the federal guidelines governing supervised injecting, as well as the stipulations of the exemption granted to the SIF [7] and stems from the potential for criminal and civil liability from assisted injection [8]. Therefore, IDU who require assistance with injection, including IDU with physical disabilities, are unable to benefit from this service. In turn, there is concern that these individuals are left to obtain assistance with their injections in unsafe injecting environments, including public and unhygienic settings such as alleyways [9]. Furthermore, research has consistently demonstrated the high risks associated with assisted injection such as increased syringe sharing [10,11], non fatal overdose [12], and elevated HIV incidence [10,13].

In an effort to address the severe harms experienced among IDU who continue to require assistance with their injections in public settings, the Vancouver Area Network of Drug Users (VANDU), a drug-user led organization, formed the Injection Support Team (IST). The IST responds to the unique needs of this population by providing peer-based education and support on safer injection practices, referring IDU to nearby social and health-related services, as well as distributing sterile injecting paraphernalia via conventional outreach methods. To inform the activities of the IST, a community-based research partnership was developed between VANDU and the British Columbia Centre for Excellence in HIV/AIDS. As part of this collaborative effort, we undertook the following analyses to examine the prevalence of assisted injection in outdoor venues, as well as the characteristics associated with those engaging in this practice.

## Methods

### Community-based research project

Since 2005, the VANDU IST has engaged with individuals who require assistance with injection or who are injecting unsafely outdoors. All IST members have been injecting for at least 10 years and have experience providing assisted injections (i.e., "hit doctors") in the DTES. There are no medical personnel on the IST. Through monthly meetings with the IST, our research team engaged in face-to-face discussions with IST to help define our study question and select variables for examination. Several members nominated by the IST were subsequently consulted to provide their expertise regarding the interpretations of the study findings, which helped navigate our selection of supporting literature for the discussion.

### Vancouver Injection Drug Users Study (VIDUS)

The following analyses are derived from the Vancouver Injection Drug Users Study (VIDUS). VIDUS is an open prospective study that has followed 1603 IDU recruited through self-referral or street outreach from Vancouver's DTES since May 1996. The cohort has been described previously in detail [14,15]. Briefly, individuals were eligible for participation if they were 14 years of age or older, had injected illicit drugs at least once in the month prior to enrolment, resided in the Greater Vancouver area and provided written informed consent. At baseline and semi-annually, participants complete an interviewer-administered questionnaire, which elicits demographic data, and information regarding drug use, injection practices, sexual risk behaviours, and enrolment into addiction treatment. Participants also provide venous blood samples, which are tested for HIV and HCV antibodies. All subjects receive a $20 stipend at each visit to compensate for their time and cover transportation costs to the facility. This study has been approved by the University of British Columbia's Research Ethics Board.

### Statistical Analysis

Our analyses examined the prevalence and correlates of reporting assisted injection in outdoors settings. Our outcome was based on the question "In the past 6 months, has anyone ever helped you to inject outdoors (i.e., street or alley)?" All participants who were currently injecting and had at least one follow-up visit between January 2004 and December 2005 were eligible for inclusion in the present analysis. Independent variables of interest included socio-demographic information: age (per year older), sex (female vs. male), Aboriginal ethnicity (yes/no), DTES residence (yes/no), homelessness (yes/no) and HIV status (yes/no). Homelessness was defined as having no-fixed address (NFA) or living on the street, in a shelter or hostel. Drug use variables of interest included: years injecting (per year), police presence (yes/no), daily heroin injection (yes/no), daily cocaine injection (yes/no), incarceration (yes/no), and involvement in the sex trade (yes/no). Police presence refers to being affected in terms of where an individual buys or uses drugs. Unless otherwise noted, all behavioural variables, both dependent and independent, refer to the six-month period prior to the interview

We examined the prevalence of receiving assistance with injection outdoors and examined factors potentially associated with reporting this practice during follow-up. As the analyses of factors correlated with assisted injection outdoors during the study period included numerous observations per participant, generalized estimating equations (GEE) were used for binary outcomes with a logit link to determine factors independently associated with our outcome throughout the follow-up period (i.e., January 2004-December 2005). These methods provided standard errors adjusted by multiple observations per person using an exchangeable correlation structure [16]. This approach also accommodates changes in predictor variables over time. As a first step, variables potentially associated with reporting assisted injection outdoors was examined in bivariate GEE analyses. To determine independent predictors of this outcome, we fit a multivariate logistic GEE model using an *a priori *defined model building protocol that involved adjusting for all explanatory variables that were found to be statistically significant at the *p *< 0.05 in bivariate analyses. All statistical analyses were performed using SAS software version 8.0 (SAS, Cary, NC).

## Results

In total, 620 participants were actively injecting and had at least one follow-up visit between January 2004 and December 2005 and thus were eligible for inclusion in the present analysis. The median age of the sample was 31.9 (Interquartile range 25.4-39.3), 251 (40.5%) participants were female, and 203 (32.7%) self-identified as Aboriginal.

The proportion of VIDUS participants who reported assisted injection outdoors varied with each follow-up between 2004 and 2005 and ranged between 8% and 15%. Univariate and multivariate results are displayed in Table 1. In multivariate analyses, assisted injection outdoors was positively associated with being female (Adjusted Odds Ratio (AOR) = 1.74, 95% Confidence Intervals (CI): 1.21-2.50), daily cocaine injection (AOR = 1.70, 95% CI: 1.29-2.24), and sex trade involvement (AOR = 1.44, 95% CI: 1.00-2.06). Aboriginal ethnicity remained negatively associated with the outcome (AOR = 0.58, 95% CI: 0.41-0.82).

**Table 1:** Socio-demographic and behavioural factors associated with reporting requiring help injecting outdoors among participants of the Vancouver Injection Drug User Study

Variable	Requiring help injecting outdoors n = 163	
	Odds Ratio (OR) (95% CI)	Adjusted OR (95% CI)
**Age**		
(year older)	1.06 (1.04-1.09) **	0.98 (0.95-1.01)
**Years injecting**		
(per year)	0.97 (0.95-0.99)**	1.00 (0.98-1.01)
**Sex**		
(female vs. male)	2.80 (1.87-4.20) **	1.74 (1.21-2.50)*
**Aboriginal ethnicity**		
(yes vs. no)	1.07 (0.70-1.64)	0.58 (0.41-0.82)*
**DTES residence**		
(yes vs. no)	1.40 (0.96-2.06)	-
**HIV**		
(yes vs. no)	1.02 (0.67-1.57)	-
**Homeless**		
(yes vs. no)	1.88 (1.22-2.90) **	1.24 (0.75-1.79)
**Daily heroin**		
(yes vs. no)	2.35 (1.65-3.34) **	1.25 (0.95-1.66)
**Daily cocaine**		
(yes vs. no)	1.45 (1.05-2.01) *	1.70 (1.29-2.24)*
**Sex trade**		
(yes vs. no)	2.85 (1.91-4.26) **	1.44 (1.00-2.06)*
**Incarceration**		
(yes vs. no)	1.82 (1.18-2.80) **	1.24 (0.87-1.77)
**Police presence**		
(yes vs. no)	2.35 (1.64-3.37)**	1.22 (0.91-1.65)

Note: * *p *< 0.050 ** *p *< 0.001, CI = Confidence Interval

## Discussion

In our study, between 8 and 15% of local IDU reported receiving assistance with injecting in outdoor settings and this practice was independently and positively associated with being female, daily cocaine injection, and sex trade involvement. Aboriginal ethnicity was negatively associated with reporting assisted injection outdoors. Given that assisted injection has been shown to be independently associated with syringe sharing [10,11] and is a risk factor for HIV infection [10,13] and overdose [12], these findings indicate that novel programs are needed to target the distinct needs of this subpopulation of IDU who engage in this practice in outdoor venues.

In the present study, we demonstrated that being female was associated with receiving assistance with injecting in outdoor settings. This finding is consistent with previous literature that demonstrates females are overrepresented among those that require assistance with their injections [10,13,17]. Females likely require help with injecting for different reasons than men; specifically, females are more likely to report that they do not know how to inject themselves [18]. Based on this finding, a gender-sensitive approach may be needed to ensure that when members of the IST approach females injecting outdoors, they are offered effective and appropriate education and advice on how to self-inject safely.

In the present study, reporting assisted injection outdoors was associated with daily cocaine injection. There is a dearth of information on the relationship between assisted injection outdoors and frequent cocaine injection. However, the aspect of binge drug use as it relates to daily cocaine injection may offer some insight. Due to cocaine's short half-life, there is a need to inject more often (e.g., 20 times a day) in order to maintain a high [15]. During periods of binge drug use, individuals can become highly stimulated, be more likely to hang out in the open drug scene, and experience sleep deprivation [19], and therefore may have reduced ability to self-administer injections. Often individuals have preference about who provides assisted injection but preferences shift during periods of drug withdrawal or availability [18], which may result in a variety of people providing assistance with injections. Further, cutaneous injection-related infections (CIRI), such as abscesses and cellulitis, can result in vascular damage, which may impair the ability of IDU to administer their own injections. Such infections have been also associated with frequent cocaine injection [20,21]. In addition, daily cocaine injection remains a strong predictor of HIV risk among IDU highlighting vulnerability in this population [15,22].

Importantly, sex trade involvement was associated with reporting assisted injection outdoors, and this association was independent from the association of female sex. When drugs are shared among sex workers and their clients, some clients are assuming responsibility for the preparation and administration of drugs [23]. Further, in our setting, Shannon *et al. *recently demonstrated that individuals involved in sex trade work are being pushed to work and inject in remote outdoor locations due to heavy police presence and laws that prevent sex workers from working in regulated indoor sex work venues [24]. The displacement of sex work into outdoor settings may explain the association between sex work and outdoor assisted injection locally.

Our results support further development of gender-based interventions that build personal capability to self inject. These initiatives are currently supported by the SIF and the IST, but their role could improve if the capacity of these services was increased. The SIF has been described as a setting in the DTES where IDU can obtain safer injection education [25]. Further, the SIF has been able to attract female injectors and individuals who require assistance with injection for CIRI care [25,26]. Importantly, drug user led organizations have been emerging globally and have demonstrated that drug users can organize themselves and make valuable contributions to their communities [27]. In particular, VANDU (all IST members are VANDU members) performs a critical education function by exposing outsiders to the realities of daily life for drug users in Vancouver's DTES [27]. Drug related harm, including risk of bacterial and viral infections, overdose, theft, and missed injection has been extensively documented among those who require assistance with injection [10,11,13,18]. Therefore, increasing number and types of services offered by the IST, who do not receive compensation for the injection related support they provide, could reduce the drug related harm in this setting. In the absence of round the clock SIF operation and to ensure remote outdoor access to clean injection supplies, injection paraphernalia vending machines may be considered as further novel intervention. In addition, the dynamic of ingrained injection routines and assisted injection by intimate partners or clients of sex trade workers [17,18,23] need to be acknowledged and considered when developing interventions (e.g., education material or individual instruction of safer injection practices) specific to females and sex trade workers. Importantly, further research is required to elucidate why Aboriginal ethnicity was the only variable negatively associated with requiring assistance with injection in outdoor settings.

There are limitations of this study to be considered. VIDUS is not a random sample. Therefore, findings from this analysis are not necessarily generalizable to the wider population of IDU in our setting or elsewhere. However, research has suggested that the VIDUS cohort is representative of IDU in the DTES community [28]. Our finding may also not be generalizable to cities with different climates from Vancouver. Additionally, since our study relied on self-report data regarding drug and injecting practices, our analysis could be subject to social desirability bias. However, other studies have suggested self-report among IDU to be valid [29]. Finally, unmeasured factors predictive of high-risk activity among IDU, including social network dynamics and membership in a large socio-metric risk network [30], may have also contributed to the observed findings but are not incorporated into our analysis. Other potential explanatory factors specific to the outdoor injecting environment, such as lack of a physically clean space and inadequate lighting [9], were not considered and may be better understood through qualitative investigation.

## Conclusions

There are important implications of the findings from the present study. It is recommended that the regulations at the SIF be changed to allow individuals who require assistance with their injection to inject at the SIF. These findings highlight the importance of ensuring that peer-based outreach programs have strong female representation as a means of ensuring that the unique needs of female IDU are addressed. It may also be important for the IST to target more remote outdoor areas that are frequented by sex workers. Furthermore, given the binge nature of cocaine injection, it would be valuable to offer SIF and IST services 24 hours a day.

Receiving assistance with injecting in outdoor settings was reported by 8 to 15% of local IDU over time. In the present study, individuals who reported assisted injection outdoors were more likely to be female, daily cocaine injectors, and individuals involved in the sex trade, and were less likely to be Aboriginal. Our findings have implications for the role of peer education and outreach programs run by drug users. This study points to the need for a broad set of interventions, such as housing and treatment initiatives, which complement current harm reduction services to reduce the levels of unsafe injecting occurring outdoors in our setting.

## Competing interests

The authors declare that they have no competing interests.

## Authors' contributions

ELS, BR and TK conceived the study. ELS and BR coordinated and designed the study. KL analyzed the data. ELS drafted the manuscript. All authors assisted in interpretation of findings or revisions for intellectual content and have given final approval of the manuscript.
